# Genetically Encoded Fluorescent Redox Probes

**DOI:** 10.3390/s131115422

**Published:** 2013-11-11

**Authors:** Wei Ren, Hui-Wang Ai

**Affiliations:** Department of Chemistry, University of California, 501 Big Springs Road, Riverside, CA 92521, USA; E-Mail: wren001@ucr.edu

**Keywords:** redox signaling, fluorescent protein (FP)-based sensors, reaction-based probes, unnatural amino acids (UAAs), redox probes

## Abstract

Redox processes are involved in almost every cell of the body as a consequence of aerobic life. In the past decades, redox biology has been increasingly recognized as one of the key themes in cell signaling. The progress has been accelerated by development of fluorescent probes that can monitor redox conditions and dynamics in cells and cell compartments. This short paper focuses on fluorescent redox probes that are genetically encoded, and discusses their properties, molecular mechanism, advantages and pitfalls. Our recent work on reaction-based encoded probes that are responsive to particular redox signaling molecules is also reviewed. Future challenges and directions are also commented.

## 1. Introduction

Under most conditions, living organisms on our planet use aerobic respiration to generate energy, in which process reactive oxygen species (ROS) are inevitably and continuously generated [1]. It is not surprising that cells have adopted a complicated system to maintain redox homeostasis, sense redox changes, and use redox chemistry to initiate, transfer and amplify biological signals. Many redox-active molecules including ROS, reactive nitrogen species (RNS), and some other redox modifiers (e.g., hydrogen sulfide (H_2_S)) can diffuse inside cells and across cell membranes, and are increasingly recognized for their important functions in cell signaling [1–4]. They interact with diverse cellular targets, leading to alterations in their oxidation states and biological functions. In many cases, multiple signaling molecules are generated to interact with the same cellular target in a competitive or synergistic manner. Due to the inherent complexity, a large part of redox homeostasis and signaling remains elusive [4].

To examine redox signaling in the cellular and molecular level, a current research focus is to develop methods to identify and characterize molecular products (e.g., modified macrobiomolecules or small molecule byproducts) resulting from redox biochemical reactions [5]. Another focus is to directly investigate signaling molecules that are actively involved in redox processes. Typically, redox signaling molecules are highly diffusible and reactive, so their detection has been a long-time challenge [6,7]. Colorimetric, electrochemical, and chromatographic assays have been explored. However, these methods often require sample processing, and do not provide much spatial and temporal information about living cells and organisms [8–10]. In addition, many redox signaling molecules, such as nitric oxide (NO) and peroxynitrite (ONOO^−^), have very short lifetimes, so it is essentially impossible to directly measure them in processed samples [11,12]. The need to reliably detect redox signaling has promoted the emergence of a group of fluorescent redox probes that can be introduced into living cells and organisms. In previous studies, a large number of synthetic probes have been designed and synthesized [7,13–16]. These molecules are diverse in structure and show different degrees of sensitivity and selectivity. When loaded into living cells, they could change their fluorescence in response to redox dynamics. Another approach is to use redox probes that are genetically encoded. Genetically encoded probes can be introduced into living cells or organisms in the format of DNA, and next, get expressed into proteins by intracellular machineries. The advantage is that encoded probes can be readily localized to specific cell compartments using corresponding localization sequences or to the vicinity of proteins of interest by creating genetic fusions [17]. Such versatility allows the investigation of biochemical dynamics with subcellular spatial resolution.

Since the discovery and cloning of green fluorescent protein (GFP) and its homologs [18,19], a considerable amount of research has been conducted to create fluorescent protein (FP)-based encoded probes that change their fluorescent color or intensity in response to external stimuli or physiological changes [20–23]. In particular, a number of FP-based probes that can sense cellular redox dynamics have been developed. These genetically encoded sensors can provide real-time and *in situ* information, and have greatly facilitated research in redox biology [24]. In this short review, we summarize common genetically encoded fluorescent probes, including those that can monitor the intracellular redox potential and particular redox signaling molecules such as hydrogen peroxide (H_2_O_2_), organic hydroperoxide (ROOH), NO, hydrogen sulfide (H_2_S) and ONOO^−^.

## 2. Redox-Active Fluorescent Proteins

**Figure 1.**
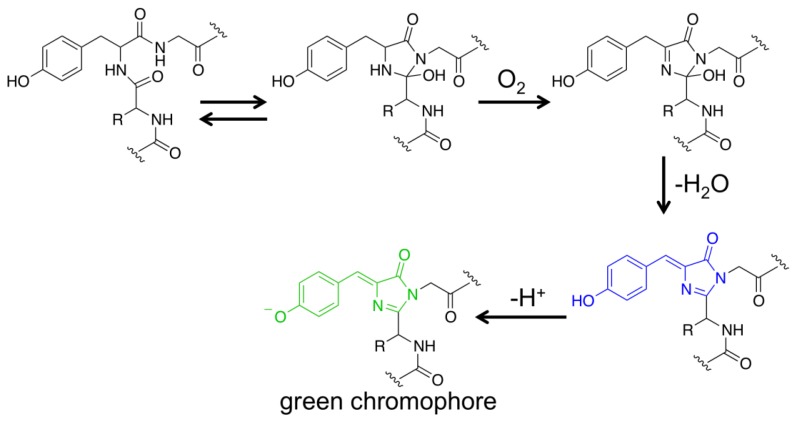
A possible pathway to form a mature green fluorescent chromophore from three residues in a GFP polypeptide.

FPs have become one of the most important research tools in biology [18,19]. Except for their genetic encodability, these proteins have a rather unique property that expression of their genes in cells or organisms is adequate to generate chromophores that are highly fluorescent in the visible spectral region. Molecular oxygen (O_2_) is the only auxiliary factor for conversion of a nascent FP polypeptide into a folded β-barrel structure containing a mature chromophore. Taking the wild-type *Aequorea victoria* GFP as an example, its Ser65, Tyr66 and Gly67 residues spontaneously undergo sequential posttranslational reactions to form a *p*-hydroxybenzylideneimidazolidone chromophore locating in the center of its β-barrel structure (Figure 1) [18,25,26]. Due to their favorable features, FPs have become popular protein scaffolds, from which generated are a large number of protein sensors that can actively change fluorescence in response to the environment [20–23].

**Figure 2.**
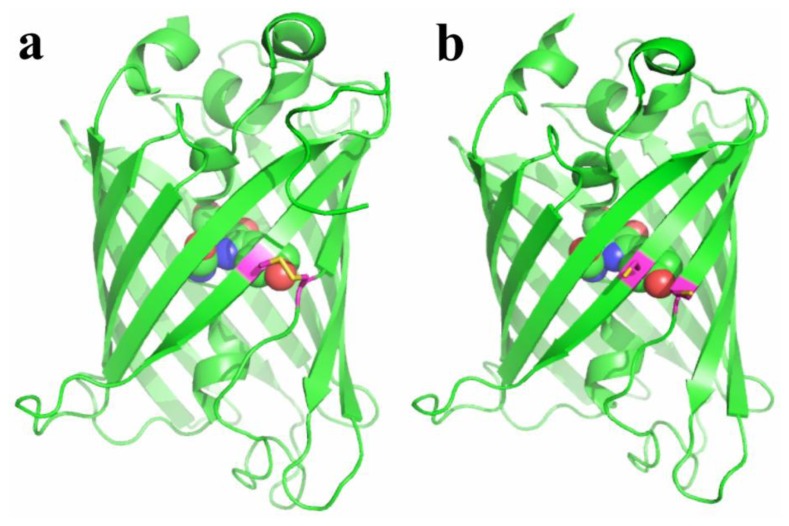
X-ray structures of roGFP variants in their oxidized (**a**) and reduced (**b**) forms (redrawn from PDB 2AH8 and 2AHA).

Redox-active FPs were generated by introducing surface-exposed cysteines residues into the β-barrels of FPs (Figure 2) [24,27,28]. The residue positions were selected so that they are in the vicinity of the chromophores. Reversible disulfide bonds between cysteines can form in response to oxidation. The oxidation status of the probes is affected by cellular environment, which in turn alters the fluorescence of FPs. The resulting probes, redox-sensitive yellow FP (rxYFP) and redox-sensitive GFP (roGFP), when expressed in cells, can respond to oxidative stimuli, mainly through a glutaredoxin (Grx)-catalyzed mechanism [29,30]. It was shown that the direct reaction between the probes and H_2_O_2_ is kinetically disfavored, and their direct equilibration with the cellular glutathione pool is also slow. However, fluorescence change is fast in the presence of Grx. That being said, rxYFP and roGFP are good sensors for the glutathione redox potential when Grx is present with sufficient concentrations in the cell type or cell compartment of interest. To gain good response and selectivity under broader conditions, a strategy of linking rxYFP and roGFP with Grx enzymes has been developed [31,32]. The resulting fusion probes showed fast equilibrium with the oxidized/reduced glutathione (GSSG/GSH) redox pair. Grx works as an enzyme to transfer glutathione *via* its *S*-glutathionylated cysteine to rxYFP or roGFP, which are next rearranged to form disulfide bridges in the FP scaffold. The aforementioned probes do not directly sense ROS (e.g., H_2_O_2_ or ONOO^−^), and fluorescent changes are only observed when the generated ROS are able to shift the GSH/GSSG equilibrium. Variants of roGFP have also been developed to show different redox potentials, which are particularly valuable for imaging redox dynamics in cell compartments with different basal redox levels [33]. roGFP is excitation-ratiometric, so it is less sensitive to the expression levels of the probe and fluorescence photobleaching, leading to better control for quantitative measurement. Indeed, roGFP is used more often than rxYFP. It is worth noting that, for both roGFP and rxYFP, the oxidation and reduction modulate the equilibrium of their chromophore between a neutral and an anionic state. Therefore, these probes are intrinsically sensitive to pH changes and additional caution is needed when interpreting fluorescence results.

## 3. Molecular Hybrids of Fluorescent Proteins and Redox Sensory Proteins

In order to directly sense H_2_O_2_, roGFP has been linked to a H_2_O_2_-specific peroxidase Orp1 [34]. H_2_O_2_ can generate an intramolecular disulfide bond in Orp1, which is next quickly transferred to roGFP through a thiol-disulfide exchange mechanism. Oxidation of Orp1 by H_2_O_2_ can be near-stoichiometrically converted to oxidation of roGFP. The oxidized roGFP-Orp1 probe is reversible in cells by reducing molecules such as thioredoxin (Trx) and potentially the Grx/GSH system. So the roGFP-Orp1 fusion responds to a balance between H_2_O_2_-induced oxidation and cell reduction.

Another approach to sense H_2_O_2_ is to directly conjugate circularly permuted FPs with redox-active protein domains, in such a way that the conformation changes in the domains can modulate the FP fluorescence. For example, a circularly permuted YFP (cpYFP) has been fused with a cysteine-containing *E. coli* OxyR regulatory domain to create a “HyPer” sensor for H_2_O_2_ [35]. Different from roGFP-Orp1, there is no redox relay in HyPer. Instead, two cysteines of OxyR can form a reversible disulfide bond, which conformational change is transferred to cpYFP and impacts its chromophore ionization states. HyPer is insensitive to many other oxidants such as superoxide (O_2_^•−^), GSSG, NO, and ONOO^−^. HyPer has been used to detect *in vitro* H_2_O_2_ in the nanomolar range. When expressed in cells, it can respond to micromolar H_2_O_2_ added to cell culture media. For live-cell imaging studies, both roGFP-Orp1 and HyPer have similar sensitivity to H_2_O_2_, but the response of roGFP-Orp1 is somewhat slower [34]. One particular caution is that, when illuminated with blue light, a portion of HyPer molecules with a deprotonated chromophore would be forced into a nonfluorescent dark state, so it is always important to distinguish H_2_O_2_-induced fluorescence changes from photobleaching [36]. Recently, further improved mutants of HyPer have been reported, and these new variants have been shown to exhibit expanded dynamic range and faster redox kinetics [37,38].

Similar to HyPer, Chen *et al.* reported a sensor for organic hydroperoxides. The probe OHSer was created by insertion of a cpYFP into the oxidative-responsive region of a transcriptional regulatory protein OhrR [39]. OhrR is a bacterial regulatory protein specific for organic hydroperoxides. OhrR is highly selective, and thus, the resulting OHSer probe is able to effectively discriminate organic hydroperoxides from other cell-generated ROS including H_2_O_2_. Both HyPer and OHSer are reversible in cells. Therefore, intracellular reducing systems may also shift the oxidation and reduction balance of HyPer or OhrR, [40] even if there is no change in the concentrations of H_2_O_2_ or organic hydroperoxides. In addition, like roGFP and rxYFP, HyPer and OhrR are sensitive to pH changes.

A few additional encoded probes were based on Förster resonance energy transfer (FRET), a distance-dependent energy transfer mechanisms between two chromophores [41]. Cysteine-rich redox-sensitive peptides were inserted between a cyan FP (CFP) and a YFP. The oxidation of cysteines would generate disulfide bonds to reduce the distance between CFP and YFP [42]. Usually, fluorescence changes of these probes are modest and slow. It is also true that many cell endogenous molecules also interfere with the process. In one example, the redox sensitive domain of bacterial hsp33 was utilized to create a HSP-FRET sensor, which was expressed in cardiomyocytes to detect redox changes [43]. In another example, cysteine-rich metallothionein has been linked to FPs [44]. The probe was shown to respond to NO, despite the expectation of low specificity as metallothionein can interact with many other intracellular molecules (e.g., metal ions, and oxidative and reducing species). Similarly, there are reported sensors based on cysteine-rich peptide or proteins for metal ions such as Zn^2+^ and Cu^+^, and we expect that these probes should also interact with redox signaling in cells [45,46].

## 4. Reaction-Based Unnatural Amino Acid-Derived Fluorescent Proteins

Cysteine is the most redox-sensitive residue among the 20 natural amino acids, and all abovementioned encoded redox probes are actually based on the reactivity of cysteine residues. In one hand, these probes are reversible so that they can reflect the dynamics of intracellular redox changes. On the other hand, each probe responds to more than one process. In particular, if both reducing and oxidizing molecules are co-generated, their responses would become very sophisticated, which may even render the use impossible. Furthermore, the limitation posed by cysteine chemistry and the availability of redox sensory proteins has put another restriction on the development of genetically encoded redox probes. Encodable sensors for many important redox-active molecules have not yet been made, merely because appropriate sensory domains are not available.

To further expand the strategies for creation of fluorescent redox probes, our laboratory explored a new method that combines unnatural amino acids (UAAs) and FPs. The introduction of UAAs into FPs by a genetic code expansion technology (reviewed in detail previously [47]) has resulted in initial success. In particular, we have shown that selective reaction-based probes for H_2_S and ONOO^−^ can be developed using such new strategy [48,49]. A comparison of these genetically encoded redox probes is shown in Table 1.

### 4.1. A Genetically Encoded Probe for Hydrogen Sulfide

H_2_S is a gaseous molecule, which is poisonous at high concentrations but also now considered an important redox signaling molecule for regulating cardiovascular, neuronal, immune, endocrine, and gastrointestinal systems [2,50]. In mammalian cells, H_2_S can be endogenously synthesized *via* both enzymatic and nonenzymatic pathways [2,51]. Cystathionine-γ-lyase (CSE) and cystathionine-β-synthetase (CBS) are responsible for the majority of endogenous H_2_S production, which converts cysteine and homocysteine to H_2_S. In addition to endogenous production, H_2_S is a metabolic byproduct of gut bacteria. H_2_S is shown to interact with metal centers in proteins. It may also interact with protein cysteine residues, resulting in *S*-sulfhydration of proteins through a still unclear mechanism [52]. The diverse biological functions of H_2_S and its potential therapeutic applications have motivated the development of new methods to monitor its production, location, trafficking and transformation in cells, tissues and whole organisms [6,14,15,53,54].

**Figure 3.**
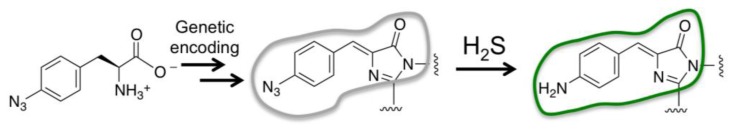
Illustration of the mechanism of a genetically encoded sensor for H_2_S.

Previous studies showed that when an azide functional group (-N_3_) was fused to conjugation systems of fluorescent dye molecules, it could decrease the fluorescence of the dyes [16,55]. The azide weakly withdraws electrons from the extended π-systems of dye molecules. When it is reduced by H_2_S to amine (-NH_2_), it donates electrons to afford a typical push–pull chromophore for greatly enhanced fluorescence. This strategy has been employed to create a group of fluorescent probes for H_2_S [16,55]. Our laboratory employed the same strategy and developed the first genetically encoded probe for H_2_S (Figure 3) [48]. A genetic code expansion technology was used to replace the chromophore-forming Tyr67 residue of a monomeric teal fluorescent protein (mTFP1) with *p*-azidophenylalanine. After the protein was synthesized in cells, a *p*-azidophenylalanine derived chromophore was formed. The resulting chromophore was reactive to H_2_S and an increased fluorescence was observed. However, the reaction was very slow since the chromophore is encapsulated in the β-barrel structure. To solve the kinetics problem, we exploited FPs in nonnative topologies. A circularly permuted GFP was constructed and its chromophore-forming Tyr66 was also replaced with *p*-azidophenylalanine. The chromophore of the resulting protein is less encapsulated, and thus, it reacted much faster with H_2_S. The resulting probe, named hsGFP later, also has very good selectivity since it was essentially unreactive to other reducing and oxidizing molecules in cells. A 1.6-fold fluorescence enhancement was observed when the probe was incubated with 100 μM H_2_S. The probe has been introduced into mammalian cells for detection of H_2_S. The probe can be genetically encoded, so it offers many advantages, such as the addition of cell localization tags to allocate the probe to specific cell subdomains. This study was also the first example of reaction-based UAA-derived FP sensors. Major limitations of this probe are its small dynamic range and incomplete maturation of the UAA-derived chromophore. Both problems are expected to be addressed in continuing work, in which directed protein evolution will be employed to further enhance properties of this H_2_S sensor.

### 4.2. Genetically Encoded Probe for Hydrogen Peroxide and Peroxynitrite

**Figure 4.**
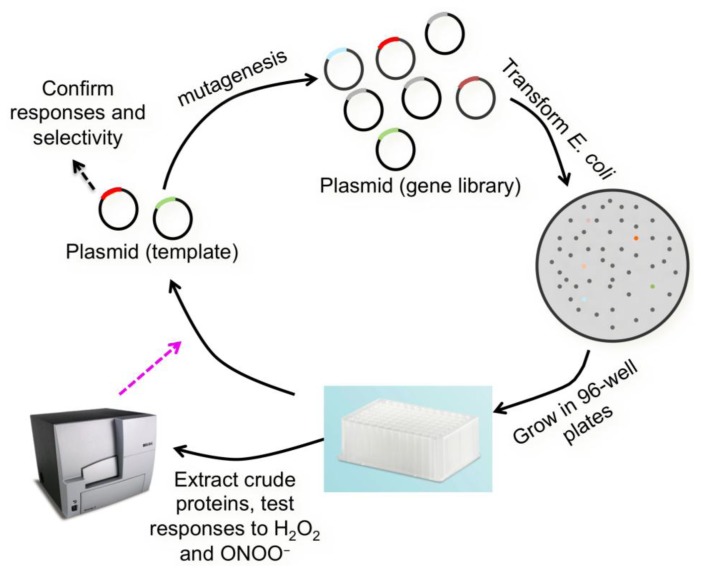
Illustration of the protein engineering steps to derive a selective probe for ONOO^−^.

Along the same line, *p*-boronophenylalanine has also been introduced into FPs to form UAA-derived chromophores. In one example, Wang *et al.* inserted *p*-boronophenylalanine into GFP in its native topology [56]. The resulting protein was expressed in *E. coli* and responded to high millimolar H_2_O_2_ added to bacterial cells. Since our work with the H_2_S sensor clearly demonstrated the advantage of using circularly permuted FPs [48], we directly incorporated *p*-boronophenylalanine into cpGFP [49]. However, such unnatural amino acid mutation was detrimental to the protein, leading to impaired protein folding and low solubility. To solve the problem, we performed direct evolution to improve cpGFP. After deriving the enhanced cpGFP2, *p*-boronophenylalanine was introduced to generate a UAA-derived FP. The probe was reactive to H_2_O_2_ in high micromolar concentrations. Previous studies showed that boronic acid could react with several different types of oxidants. So we tested our probe against a panel of ROS. Indeed, we found that our probe was also responsive to O2^•−^ and ONOO^−^. Thus, an encoded sensor for a collection of ROS was developed. At the same time, we also converted a superfolder GFP [57] into a circularly permuted protein cpsGFP. *p*-Boronophenylalanine was also introduced into cpsGFP, and both cpGFP2 and cpsGFP were used as our templates in the following protein engineering steps (Figure 4) to derive selective sensors for particular ROS. Briefly, gene libraries of cpGFP2 and cpsGFP were generated. Crude *p*-boronophenylalanine derived proteins were extracted and subjected to a screening assay against multiple ROS. We were fortunate to identify a mutant that can selectively respond to ONOO^−^, but not other reducing or oxidizing reagents at physiologically relevant concentrations [49]. The probe was named pnGFP. It can detect ONOO^−^ influx down to tens of nanomolar per min. To utilize pnGFP in mammalian cells, we further employed a fluorescence assay to screen orthogonal aminoacyl tRNA and synthetase pairs previously developed for mammalian encoding of other UAAs. We identified a polyspecific synthetase and used it to express pnGFP in human embryonic kidney (HEK) 293T cells. Selective imaging of ONOO^−^ in living HEK 293T cells was achieved.

The reason for the extraordinary selectivity of pnGFP is still not elucidated. Several residues surrounding the boronic acid-derived chromophore are Ser or Thr. Presumably they can interact with the unnatural chromophore and convert sp^2^-hybridized boronic acid into boronic ester or even sp^3^-hybridized structures. Studies are currently being pursued to elucidate the mechanism.

pnGFP was the first genetically encoded probe for ONOO^−^, a less-studied redox-active molecule formed *in vivo* from the diffusion-controlled reaction between O2^•−^ and NO (k = 6.7 × 10^9^ M^−1^ s^−1^) [11]. ONOO^−^ can function as an oxidant and also an efficient nitration reagent in cells [58]. Misregulated oxidation and nitration of biomolecules by ONOO^−^ is detrimental and has been linked to Alzheimer's, arthritis, cancer, autoimmune and inflammatory diseases, and other disorders [59]. In normal physiological conditions, biomolecules may also be oxidized or nitrated for the purposes of signal transduction and immunogenic response. In that context, the first genetically encoded ONOO^−^ probe is expected to enable new studies about the dynamics of ONOO^−^, leading to advance in a broad field of redox chemistry and biology. The study also demonstrated a unique advantage of protein-templated reaction-based probes: directed protein evolution is powerful in deriving selectivity that is hard to gain merely from rational design.

## 5. Perspectives

FP-based redox sensors can be targeted to individual cell compartments or genetically fused to proteins of interest. They have been utilized to study biochemical dynamics in cells and at subcellular locations. Such technical advancement has greatly accelerated research in redox biology. Despite the progress, all current encodable redox probes emit light in the green to yellow-green spectral region [24]. Therefore, monitoring redox changes in multiple compartments is technically difficult. Although the problem can be partially alleviated when researchers have the access to expensive equipment with the spectral deconvolution ability, the best and simplest solution is to develop additional color-compatible redox probes. The development would also be necessary for simultaneous measurement of multiple parameters of interest. In addition, cells generate a large group of ROS, RNS and other redox active molecules. The availability of encoded probes is currently limited to a very small number of redox-active molecules. Future work is needed to further expand the toolbox of redox probes. Selective and sensitive detection of each redox signaling molecule will be achieved to assist the elucidation of biological roles of these signaling molecules in physiology and pathology.

## Figures and Tables

**Table 1.** A list of genetically encoded redox probes. The table does not include all reported probes, but a selection based on their biological applications.

***Redox Probe***	***Main Analyte***	***Excitation Max. (nm)***	***Emission Max. (nm)***	***Comments***
rxYFP [28,29]	glutathione redox potential	512	523	Midpoint redox potential (2GSH/GSSG): −261 mV
roGFP [27,33]	glutathione redox potential	405 and 488	510	Excitation-ratiometric; mutants with various redox potentials (∼ −230 to −300 mV) suitable for studies in different cell organelles; roGFP2 is widely used
rxYFP-Grx1p [31]	glutathione redox potential	512	523	Thiol–disulfide exchange with enhanced rate of response
Grx1-roGFP2 [32]	glutathione redox potential	405 and 488	510	Excitation-ratiometric; rapid equilibration with the glutathione redox pair;
HyPer [35,37,38]	hydrogen peroxide	420 and 500	516	HyPer3 [38] has the best dynamic range and reaction kinetics; respond to H_2_O_2_-induced oxidation and cell thiol reduction
roGFP2-Orp1 [34]	hydrogen peroxide	405 and 488	510	Respond to the balance between H_2_O_2_-induced oxidation and Trx or Grx-mediated thiol reduction
OHSer [39]	Organic hydroperoxide	519	526	Respond to organic hydroperoxides and organic alkoxyl radicals, as well as cell reduction
hsGFP [48]	hydrogen sulfide	483	512	Selectively react with H_2_S; cross-react with dithiothreitol (DTT)
pnGFP [49]	peroxynitrite	484	508	Highly selective to peroxynitrite; respond to high mM H_2_O_2_
